# A multilevel analysis of determinants of PMTCT service utilisation among women during the antepartum, intrapartum and postpartum period in Ethiopia

**DOI:** 10.1186/s12884-021-03896-1

**Published:** 2021-07-03

**Authors:** Tsegaye Gebremedhin, Tesfa Sewunet Alamneh, Asebe Hagos, Beimnet Desalegn, Nigusu Worku

**Affiliations:** 1 Department of Health Systems and Policy, Institute of Public Health, College of Medicine and Health Sciences, University of Gondar, Gondar, Ethiopia; 2 Department of Epidemiology and Biostatistics, Institute of Public Health, College of Medicine and Health Sciences, University of Gondar, Gondar, Ethiopia; 3 School of Public Health, College of Medicine and Health Science, Wolaita Sodo University, Wolaita Sodo, Ethiopia

**Keywords:** PMTCT, Utilization, Multilevel, Ethiopia

## Abstract

**Background:**

Mother-to-child transmission (MTCT) is the largest source of HIV infection in children below the age of 15 years, and more than 90% of pediatric HIV are infected through mother to child transmission. Without treatment, one-half of those infected children will die before the age of 2 years. Despite this, there is limited evidence on PMTCT and its determinants. Therefore, this study aimed to determine the factors affecting the PMTCT service utilisation in Ethiopia.

**Methods:**

A two-stage stratified sampling technique was used to identify 4081 women from 2016 Ethiopian Demographic and Health Survey (EDHS). A multilevel mixed-effect binary logistic regression analysis was used to identify the individual and community level factors associated with PMTCT services utilisation. In the final model, a *p*-value of < 0.05 and Adjusted Odds Ratio (AOR) with 95% confidence interval (CI) were used to declare statistically significant factors with the utilisation.

**Results:**

Overall, 21.9% (95% CI, 20.6–23.2) of the women were utilized PMTCT services. Educational status; primary (AOR: 1.65, 95% CI: 1.27–2.13), secondary (AOR: 1.52, 95% CI: 1.03–2.24) and higher school (AOR: 2.48, 95% CI: 1.45–4.22), poorer (AOR: 1.62, 95% CI: 1.12–2.37), middle (AOR: 1.82, 95% CI: 1.10–3.02), richer (AOR: 2.44, 95% CI: 1.42–4.21) and richest (AOR: 4.45, 95% CI: 2.43–8.14) wealth status and orthodox religion follower (AOR: 1.62, 95% CI: 1.22–2.16) were the individual level factors. Moreover, having basic (AOR: 1.66, 95% CI: 1.34–2.06) and comprehensive (AOR: 1.73, 95% CI: 1.38–2.18) knowledge on HIV prevention methods, having knowledge on MTCT of HIV (AOR: 2.69, 95% CI: 2.16–3.36) were also factors at individual level. Whereas, rural residence (AOR: 0.52, 95% CI: 0.32–0.85) was the community level factors that affects the utilization.

**Conclusions:**

Less than one-fourth of the mothers had utilised the PMTCT services in Ethiopia. To increase the utilisation of the services, the health care providers should give emphases on counselling, awareness creation, and strengthen the existing frontline integrated health care services in the country.

## Background

Globally, an estimated 1.8 million children were living with the human immune deficiency virus (HIV), and about 110,000 children died from AIDS-related illness in 2015. More than 1.6 million children were living with HIV in Sub-Saharan Africa [1]. In Ethiopia, there were 62,000 children less than 15 years old living with HIV, 3800 new infections, and 2900 AIDS-related deaths among children in 2016 [2].

Mother-to-child transmission (MTCT) is the largest source of HIV infection in children below the age of 15 years [3], and more than 90% of pediatric HIV are infected through mother-to-child transmission [4]. Without treatment, one-half of the infected children will die before 2 years [5]. In the absence of any intervention, MTCT rates range from 15 to 45% [6]. The vast majority of these infections were averted between 2010 and 2015, globally, because of PMTCT service and ART initiation [7].

In August 2012, Ethiopia adopted the World Health Organization (WHO) PMTCT programmatic shift services strategy [8]. PMTCT option B + has the greatest benefits in settings with high prevalence and high fertility in which initiating ART among all pregnant and breastfeeding women would reduce HIV incidence and prevalence of transmission in both current and future pregnancies [9]. The percentage of pregnant women living with HIV who were receiving antiretroviral therapy (ART) increases from 64% in 2013 to 73% in 2014 and reducing the incidence of new child HIV infections by 65% since 2009 in Ethiopia [10]. However, only 35% of HIV infected children received ART therapy in Ethiopia [2]. A meta-analysis finding showed that the pooled prevalence of MTCT of HIV in Ethiopia was 9.93%, which means almost one in every ten HIV-exposed infants become HIV positive. Mothers who did not use the recommended PMTCT intervention during pregnancy, labor and delivery, and breastfeeding are more than seven times more likely to transmit HIV to their child [11]. The PMTCT service utilisation in Afar, Ethiopia, was reported as 67.7% [12]. Only 18 and 9% of respondents attended the facility for HIV counselling and testing (HCT) and receiving antiretroviral prophylaxis, respectively, in Addis Ababa, Ethiopia [13]. Another study in the Amhara region of Ethiopia reported that the utilisation of PMTCT service among pregnant women attending ANC was 61.3%. The utilisation of PMTCT service among ANC attendants in rural areas was 57.8%. More than half (55.5%) of the pregnant mothers did not start ANC properly [14].

Different literatures revealed that PMTCT service utilisation was affected by knowledge of PMTCT, residence, internal referral system, health professional-client interaction, fear, and waiting time, the number of ANC visits, education status, and involvement in PMTCT services. PMTCT training, lack of materials and equipment for PMTCT service, language barrier, and the low number of PMTCT providers were the major barriers identified by the PMTCT service provider in providing PMTCT service [12, 14].

Empirical studies have been conducted on PMTCT services utilisation and associated factors, but to our knowledge, there is no study conducted at the national level, which identifies the individual and community level determinants of PMTCT services utilisation. Thus, this study aimed to determine the individual and community level factors of PMTCT services utilisation among women during antepartum, intrapartum, and postpartum periods in Ethiopia from the 2016 Ethiopian demographic and health survey. This study may provide additional information by identifying the individual and community level factors that improve the PMTCT service.

## Methods

### Study settings and data source

The study was carried out in Ethiopia, which is the second populous country in Africa, with a population density of 115 people per Km^2^ [15]. Ethiopia is divided into two administrative units (Addis Ababa and Dire Dawa) and nine regions (Tigray, Afar, Amhara, Oromia, Somali, Benishangul, SNNPR, Gambella, and Harari).

This analysis used the 2016 Ethiopian Demographic and Health Survey (EDHS) data, which is a nationally representative household survey data conducted every 5 years by Ethiopia’s Central Statistical Agency (CSA) [16].

### Sampling procedures and sample size

In the survey, all women aged 15 to 49 who are regular members of the selected households were included. Finally, data from the 2016 EDHS datasets were analysed using STATA version 14 software, and a total weighted of 4081 women were included in the final analysis. Individual and community level independent variables were identified and further analysis was done.

### Measurements of variables

The study’s dependent variable was PMTCT utilization. PMTCT utilization was measured as a dichotomous variable; when a pregnant woman received all the components of PMTCT services (pre and post-test counselling, HIV testing, and receiving test results) during each of the three phases of the visit (antepartum, intrapartum, and postpartum), the utilisation was measured as ‘YES’, otherwise ‘NO’ [4, 6, 9, 17]. The information was gathered from mother’s verbal responses; during ANC visits, labor and delivery, and postnatal care.

Explanatory variables included the individual and community levels variable. Both maternal (socio-demographic and maternal health-care-related characteristics) and child-related variables were included in the individual level variables. At the same time, community-level variables included place of residence, region, distance to a health facility, community-level poverty, women empowerment and media exposure.

Distance to a health facility was assessed by the question “distance to the nearest health facility is a problem?” and the responses were categorised as “big problem” or “not a problem”.

Women empowerment was measured by their decision-making power and the justification for wife-beating. Women who were empowered were those who, in all situations, engaged in decision-making, either alone or with their husbands, and never justified wife-beating.

The asset index was used to assess community-level poverty, based on data from the entire sample on separate scores prepared for rural and urban households, and combined to produce a single asset index for all households at the community level, which was then ranked into three categories (poor, middle, and rich).

Community media exposure was assessed as “yes” if they have access to all three media (newsletter, radio, and TV) at least once a week, otherwise “no”.

### Data processing and statistical analysis

The data were extracted, cleaned, re-coded, and analysed using STATA version 14.1 software. Weighting was considered during the analysis to adjust for unequal probability of selection due to the sampling design used in EDHS data.

Since the DHS data had hierarchical nature (an individual were nested within communities), a two-level binary logistic regression model was fitted to estimate the effect of both individual and community-level variables on PMTCT service utilisation [18]. As a principle in multilevel analysis, four models were considered for the data. The first model was an empty (null) model without any explanatory variables. The second model was fitted with individual-level variables only; the third model was fitted community-level variables only and the fourth was adjusted for both individual and community-level variables. The variation between clusters was assessed by computing Intra-Class Correlation Coefficient (ICC) and Proportional Change in Variance (PCV). The ICC is the proportion of variance explained by the grouping structure in the population which computed as: $$ \mathrm{ICC}=\frac{{\sigma_{\mu}}^2}{{\sigma_{\mu}}^2+\raisebox{1ex}{${\pi}^2$}\!\left/ \!\raisebox{-1ex}{$3$}\right.} $$; where the standard logit distribution has a variance of $$ \raisebox{1ex}{${\uppi}^2$}\!\left/ \!\raisebox{-1ex}{$3$}\right. $$, *σ*_*μ*_^2^ indicates the community variance. Whereas PCV measures the total variation explained by individual and community level variables in the models as compared to the null model, which is computed as: $$ \frac{\mathrm{variance}\ \mathrm{of}\ \mathrm{null}\ \mathrm{model}-\mathrm{variance}\ \mathrm{of}\ \mathrm{full}\ \mathrm{model}}{\mathrm{variance}\ \mathrm{of}\ \mathrm{null}\ \mathrm{model}} $$ [19, 20]. Both bi-variable and multivariable multilevel binary logistic regressions were estimated and a *p*-value of less than 0.05 and an Adjusted Odds Ratio (AOR) with 95% Confidence Interval (CI) were used to declare statistically significant factors associated with PMTCT services utilisation in the selected model.

## Results

### Socio-demographic and economic characteristics of participants

A total of 4081 women were included in the final analysis. The women’s mean age was 28.0 ± 6.4 years, and 79.6% of the women were rural dwellers. The majority (35.1%) of the households were in the poorest wealth status, and the mean family size was 5.8 ± 2.3. Nearly 15.2 and 5.0% of the participants were from the Oromia region and Addis Ababa city, respectively. Half of the participants were Muslim in religion. Of the participants, 95.1% were married, 59.2% with no education, and 61.5% had no work. Similarly, 45.3 and 9.9% of their husbands/partners had no education and work, respectively (Table 1).

**Table 1:** Socio-demographic and economic characteristics of study participants in Ethiopia, 2016 (*n* = 4081)

Variables	Category	Frequency (n)	Percent (%)
Age in complete years	15–24	1279	31.3
	25–34	2035	49.9
	> = 35	767	18.8
Residence	Urban	832	20.4
	Rural	3249	79.6
Region	Tigray	443	10.8
	Afar	383	9.4
	Amhara	374	9.2
	Oromia	619	15.2
	Somali	527	12.9
	Benishangul	323	7.9
	SNNPR	489	12.0
	Gambela	268	6.6
	Harari	234	5.8
	Addis Ababa	207	5.0
	Dire Dawa	214	5.2
Religion	Muslim	2041	50.0
	Orthodox	1226	30.0
	Protestant	715	17.5
	Others^a^	99	2.5
Sex of head of household	Male	3224	79.0
	Female	857	21.0
Household wealth status	Poorest	1432	35.1
	Poorer	683	16.7
	Middle	567	13.9
	Richer	512	12.6
	Richest	887	21.7
Current marital status	Married	3882	95.1
	Unmarried	199	4.9
Educational status of women	No education	2417	59.2
	Primary education	1138	27.9
	Secondary education	347	8.5
	Higher	179	4.4
Educational status of husband’s/partner’s (*n* = 3882)	No education	1759	45.3
	Primary education	1302	33.5
	Secondary education	449	11.6
	Higher education	341	8.8
	Don’t know	31	0.8
Respondent’s occupation	No work	2510	61.5
	Professional	508	12.5
	Agricultural	736	18.0
	Daily labour	214	5.2
	Others^b^	113	2.8
Husband’s/partner’s occupation (n = 3882)	No work	385	9.9
	Professional	648	16.7
	Agricultural	2044	52.7
	Daily labour	418	10.8
	Others^b^	387	9.9

^a^others: catholic, traditional, Job ^b^merchant, self-employed

### Obstetric history of participants

The majority (53.7%) of the women were multipara, and 55.6% were in the age group of 18 to 24 years when they gave their first birth. Two-thirds of the women were received ANC visits for their recent pregnancy. Of those who had ANC visits, 55.0% had four and above times, and 52.8% were done their first antenatal care visits between the fourth and sixth months of their gestational age. Furthermore, 41.0% of the women delivered at health facilities, and 8.9% of them had postnatal checks within 2 months after delivery, in which nearly 41% of the postnatal checks’ services were provided by nurses (Table 2).

**Table 2:** Obstetric characteristics of study participants in Ethiopia, 2016 (n = 4081)

Variables	Category	Frequency (n)	Percent (%)
Age of the mother at first birth (in years)	< 18	1450	35.5
	18–24	2270	55.6
	25+	361	8.9
Parity	Primipara	881	21.6
	Multipara	2192	53.7
	Grand multipara	1008	24.7
ANC visit	No	1327	32.5
	Yes	2754	67.5
ANC visit (*n* = 2754)	Once	1239	45.0
	Four and above	1515	55.0
Timing of 1st ANC check in months (n = 2754)	1–3	1032	37.5
	4–6	1455	52.8
	7+	267	9.7
Wanted pregnancy when became pregnant	Then	3312	81.2
	Later	545	13.3
	No more	224	5.5
The desire for more children	Wants	2803	68.7
	Undecided	207	5.1
	Wants no more	1071	26.2
Place of delivery	Home	2409	59.0
	Health facility	1672	41.0
PNC check within 2 months	Yes	362	8.9
	No	3719	91.1
The person who provided PNC services (n = 362)	Doctor	56	15.5
	Nurse	148	40.9
	Midwife	31	8.6
	HEWs	110	30.4
	Others	17	4.7
Timing after delivery postnatal check took place (*n* = 362)	Within 24 h	39	10.8
	1-7th day	197	54.4
	After the 7th day	126	34.8
Currently pregnant	Yes	220	8.4
	No	3861	94.6
Current pregnancy wanted (*n* = 220)	Then	169	76.8
	Latter	37	16.8
	Not at all	14	6.4

*ANC* Antenatal care, *PNC* Postnatal care

### Health services related factors, women empowerments, and community-level factors

Of the total participants, only 5.9% were had access to all media types (radio, newsletter, and television) more than once a week. Nearly 46 % of the participants responded that the health facilities are not far. One-third of the women were empowered, and 54.8% of the community were in poor wealth status (Table 3).

**Table 3:** Health services related, community women empowerments and other community-level chrematistics of study participants in Ethiopia, 2016 (n = 4081)

Variables	Category	Frequency (n)	Percent (%)
Exposure to mass media	No	3840	94.1
	Yes	241	5.9
Distance to the health facility	Big problem	2207	54.1
	Not a problem	1874	45.9
Women empowerment (n = 3882)	Not empowered	2626	67.6
	Empowered	1256	32.4
Community-level poverty	Poor	2235	54.8
	Middle	692	16.9
	Rich	1154	28.3

### Knowledge about HIV and MTCT of HIV

Of the total respondents, 38.2% had the basic knowledge of HIV prevention methods, and only 22.4% of the women had a comprehensive knowledge of HIV. Furthermore, 52.2% of the women knew that HIV could be transmitted during pregnancy, labor and delivery, and breastfeeding.

### PMTCT services utilisation

Overall, 21.9% (95% CI: 20.6–23.2) of the women were received all the components of PMTCT services during the antepartum and intrapartum periods. Only 25.2% of the women have received all the counselling components on HIV (the counselling contains; babies getting HIV from their mother, preventing the virus, and being tested for HIV). Moreover, 26.2% of the women were tested for HIV tests, received their HIV test results, and received post-test counselling.

### Random effects (measures of variation)

There was a significant variation in the utilisation of PMTCT services among women across the clusters. The ICC in the null model for PMTCT services utilisation was 48.67%. In other words, 48.67% of the variation in PMTCT services utilisation among women was due to the differences between clusters (between-cluster variation). Also, the median odds ratio also revealed that PMTCT services utilisation was heterogeneous among clusters. It was 5.34 in the first model, which implies that women within cluster of higher PMTCT services utilisation had 5.34 times higher chance of PMTCT services utilisation than women within cluster of lower PMTCT services utilisation if women were selected randomly from two different clusters (EAs). Regarding PCV, about 73.00% of PMTCT services utilisation variability was explained by the full model. The full model was selected as the best-fitted model (had the lowest information criterion) (Tables 4 and 5).

**Table 4:** Result from a random intercept model (a measure of variation) for PMTCT services utilisation among pregnant and labouring women at cluster level by multilevel logistic regression analysis, EDHS 2016

Measure of vibrations	Model 0 (null model)	Model 1	Model 2	Model 3 (full model)
Variance	3.11	0.86	1.31	0.84
Explained variation (PCV) (%)	Ref.	72.35	57.87	73.00
ICC (%)	48.67	20.72	28.5	20.38
MOR	5.34	2.41	2.97	2.39
Model fitness				
Deviance (−2* log-likelihood)	3742.72	3122.64	3207.70	2935.6
AIC	3746.71	3176.65	3223.70	3001.60

*AIC* Akaike’s Information Criterion, *ICC* Intra-class Correlation Coefficient, *PCV* Proportional Change in Variance, Model 0: without independent variables (null model), Model 1: only individual-level variables, Model 2: only community-level variables, Model 3: individual and community-level variables (full model).

**Table 5:** Multilevel logistic regression analysis of individual and community-level factors associated with PMTCT option B+ services utilization among women in Ethiopia, EDHS 2016

Variables	PMTCT option B+		COR (95%CI)	Model 1 AOR (95% CI)	Model 2 AOR (95% CI)	Model 3 (AOR (95%CI)
	Utilized n (%)	Not utilized n (%)				
Educational status						
No education	285 (11.8)	2132 (88.2)	1	1 1.79 (1.40–2.29) 1.65 (1.14–2.39) 2.78 (1.70–4.54)		1
Primary	340 (29.9)	798 (70.1)	2.77 (2.21–3.47)			1.65 (1.27–2.13) *
Secondary	150 (43.2)	197 (56.8)	4.37 (3.14–6.08)			1.52 (1.03–2.24) *
Higher	120 (67.0)	59 (33.0)	12.48 (7.95–19.60)			2.48 (1.45–4.22) *
Wealth status						
Poorest	95 (6.6)	1337 (93.4)	1	1 1.62 (1.14–2.32) 2.17 (1.51–3.11) 3.28 (2.28–4.71) 8.52 (5.97–12.17)		1
Poorer	94 (13.8)	589 (86.2)	2.01 (1.41–2.86)			1.62 (1.12–2.37) *
Middle	99 (17.5)	468 (82.5)	2.93 (2.05–4.21)			1.82 (1.10–3.02) *
Richer	126 (24.6)	386 (75.4)	5.30 (3.70–7.59)			2.44 (1.42–4.21) *
Richest	481 (54.2)	406 (45.8)	21.22 (15.18–29.65)			4.45 (2.43–8.14) *
Religion						
Muslim	302 (14.8)	1739 (85.2)	1	1 1.80 (1.37–2.36) 1.04 (0.74–1.45) 0.91 (0.39–2.11)		1
Orthodox	447 (36.5)	779 (63.5)	3.21 (2.34–4.40)			1.62 (1.22–2.16) *
Protestant	135 (18.9)	580 (81.1)	1.84 (1.24–2.71)			0.98 (0.69–1.38)
Others	11 (11.1)	88 (88.9)	0.99 (0.42–2.32)			0.89 (0.38–2.09)
Occupation						
No work	482 (19.2)	2028 (80.8)	1	1 1.04 (0.77–1.40) 1.05 (0.79–1.41) 0.94 (0.61–1.44) 0.86 (0.48–1.54)		1
Professional	193 (38.0)	315 (62.0)	1.96 (1.48–2.61)			1.06 (0.77–1.45)
Agricultural	131 (17.8)	605 (82.2)	0.87 (0.65–1.18)			1.08 (0.81–1.46)
Daily labour	58 (27.1)	156 (72.9)	1.29 (0.84–1.98)			0.98 (0.62–1.57)
Others	31 (27.4)	82 (72.6)	1.04 (0.59–1.82)			0.93 (0.50–1.75)
Age at first birth (n years)						
< 18	250 (17.2)	1200 (82.8)	1	1 1.05 (0.84–1.31) 1.09 (0.75–1.60)		1
18–24	507 (22.3)	1763 (77.7)	1.23 (0.99–1.53)			1.03 (0.82–1.30)
25+	138 (38.2)	223 (61.8)	1.83 (1.28–2.62)			0.90 (0.59–1.37)
Parity						
Primipara	274 (31.1)	607 (68.9)	1	1 1.01 (0.78–1.30) 1.08 (0.76–1.55)		1
Multipara	492 (22.4)	1700 (77.6)	0.72 (0.57–0.90)			1.00 (0.77–1.31)
Grand multipara	129 (12.8)	879 (87.2)	0.54 (0.40–0.73)			1.10 (0.75–1.60)
Desired child						
Wants	615 (21.9)	2188 (78.1)	1	1 0.61 (0.37–1.01) 1.04 (0.82–1.33)		1
Undecided	34 (16.4)	173 (83.6)	0.62 (0.38–1.02)			0.59 (0.35–1.01)
Wants no more	246 (23.0)	825 (77.0)	0.89 (0.71–1.11)			1.01 (0.78–1.31)
Wanted pregnancy when became pregnant						
Then	725 (21.9)	2587 (78.1)	1	1 0.99 (0.75–1.30) 0.66 (0.41–1.07)		1
Later	133 (24.4)	412 (75.6)	0.98 (0.74–1.29)			1.03 (0.77–1.38)
No more	37 (16.5)	187 (83.4)	0.55 (0.34–0.88)			0.67 (0.39–1.14)
Knowledge of prevention method						
No	324 (12.8)	2199 (87.2)	1	1 1.70 (1.38–2.10)		1
Yes	571 (36.6)	987 (63.4)	3.29 (2.68–4.03)			1.66 (1.34–2.06) *
Comprehensive knowledge of HIV						
No	500 (15.8)	2668 (84.2)	1	1 1.74 (1.40–2.17)		1
Yes	395 (43.3)	518 (56.7)	3.18 (2.55–3.95)			1.73 (1.38–2.18) *
Knowledge on PMTCT						
No	217 (11.1)	1732 (88.9)	1	1 2.66 (2.14–3.29)		1
Yes	678 (31.8)	1454 (68.2)	3.73 (3.01–4.63)			2.69 (2.16–3.36) *
Residence						
Urban	430 (51.7)	402 (48.3)	1		1	1
Rural	465 (14.3)	2784 (85.7)	0.08 (0.06–0.11)		0.13 (0.09–0.19)	0.52 (0.32–0.85) *
Community wealth status						
Poor	265 (11.9)	1970 (88.1)	1		1	1
Middle	187 (27.0)	505 (73.0)	2.29 (1.73–3.02)		2.08 (1.57–2.74)	1.25 (0.86–1.82)
Rich	443 (38.4)	711 (61.6)	4.49 (3.48–5.79)		3.73 (2.89–4.81)	1.38 (0.90–2.12)
Exposure to media						
No	740 (19.3)	3100 (80.7)	1		1	1
Yes	155 (64.3)	86 (35.7)	4.79 (3.23–7.11)		1.72 (1.15–2.57)	1.37 (0.91–2.06)
Women empowerment						
Not empowered	445 (17.0)	2181 (83.0)	1		1	1
Empowered	401 (31.9)	855 (68.1)	1.70 (1.37–2.13)		1.17 (0.94–1.47)	1.10 (0.87–1.38)
Distance to the nearest facility						
Far	317 (14.4)	1890 (85.6)	1		1	1
Not far	578 (30.8)	1296 (69.2)	1.81 (1.44–2.26)		1.25 (0.99–1.57)	1.09 (0.86–1.37)

*statistically significant at *p*-value < 0.05

*AOR* Adjusted Odds Ratio, *COR* Crude Odds Ratio, *ICC* Intra-class Correlation Coefficient, *PMTCT* Prevention of Mother-to-Child Transmission, Model 1: adjusted for individual-level characteristics, Model 2: adjusted for community-level characteristics, Model 3: adjusted for both individual and community-level characteristics (full model)

### Factors affecting PMTCT services service utilisation (fixed effects)

After adjusting for individual and community level factors (model 3), women’s educational status, wealth status, religion, knowledge on HIV prevention methods, comprehensive knowledge on HIV, knowledge on MTCT, and residence were significantly associated with the utilisation of PMTCT services among women.

Accordingly, the odds of PMTCT utilisation among women who completed primary, secondary, and higher school were 1.65 (AOR: 1.65, 95% CI: 1.27–2.13), 1.52 (AOR: 1.52, 95% CI: 1.03–2.24), and 2.48 (AOR: 2.48, 95% CI: 1.45–4.22) times higher than those who did not have education, respectively. Women who are in the poorer, middle, richer, richest wealth status were had 1.62 (AOR: 1.62, 95% CI: 1.12–2.37), 1.82 (AOR: 1.82, 95% CI: 1.10–3.02), 2.44 (AOR: 2.44, 95% CI: 1.42–4.21), and 4.45 (AOR: 4.45, 95% CI: 2.43–8.14) times higher odds of PMTCT services utilisation compared to those who are in the poorest wealth status, respectively. Besides, those orthodox religion follower women were 1.62 times more utilise services compared to those who follow Muslim religion (AOR: 1.62, 95% CI: 1.22–2.16). Those women who had the basic knowledge of HIV prevention methods were 1.66 times more utilise the services compared to their counterparts (AOR: 1.66, 95% CI: 1.34–2.06) and women who had a comprehensive knowledge of HIV were utilised the services PMTCT services 1.73 times more than their counterparts (AOR: 1.73, 95% CI: 1.38–2.18). Women who had knowledge on MTCT of HIV were 2.69 times more utilised the PMTCT services than those who did not have knowledge (AOR:2.69, 95% CI:2.16–3.36). Furthermore, those rural dwellers were 48% less likely to utilise the PMTCT services compared to those urban dwellers (AOR: 0.52, 95% CI: 0.32–0.85) (Table 5).

## Discussion

The study identified the individual and community level determinants of PMTCT services utilisation in Ethiopia. Overall, 21.9% (95% CI: 20.6–23.2) of the women were utilised the PMTCT services during the antepartum, intrapartum, and postpartum periods. Women’s educational status, wealth status, religion, knowledge on HIV prevention methods, comprehensive knowledge on HIV, knowledge on MTCT, and residence were the determinants of PMTCT services utilisation.

The finding is in line with that of a study conducted in Addis Ababa, in which 18% of women utilised PMTCT services [21]. However, this result was greater than the finding of EDHS 2011 [22]. This higher finding might be because of the mother’s awareness, access to health services, health service expansion, and increased number of health professions, but lower than these of studies conducted in East Hararge Zone (72.8%) [23], Hawassa city (90.3%) [24], Afar region (67.7%) [25], Enugu, southeast Nigeria (93.4%), and Uganda (30.1%) [26], The variation might be our study was a national aggregated data.

This study’s findings show that women who were completed primary, secondary, and higher education levels were (1.65, 1.52, and 2.84) times more likely to utilise PMTCT services than those who were illiterates. This finding was supported by a study done in the Afar region [25], which revealed that being uneducated was one of the barriers to PMTCT services utilisation. This might be due to the education opportunity, contributes to PMTCT services knowledge, and also study from Kombolcha Town, South Wollo [27] shows a strong association between knowledge of PMTCT service utilisation and educational status. The possible reason might be that as the women got formal education, they can understand the importance of things better and get information from different sources, more fascinated to accept new things and practice new behaviour. So as the mother’s educational status (knowledge) increases, their concern for offspring becomes high, so they use the services to reduce its transmission to their child.

Those women in poorer, middle, richer, and richest wealth status were (1.62, 1.82, 2.44, and 4.45) times more likely to utilise the service compared to those in the poorest wealth status. This study was supported by a study conducted in Ethiopia [28] and Amhara region [12, 14]. The possible reason might be they can cover their transportation cost and they can get quality health care services either in private or public health facilities towards their interest. Those orthodox religion followers’ women were 1.62 times more utilising PMTCT services than those who follow Muslim religion; this finding was in line with a study done in Kenya [29].

Those women who had a basic knowledge of HIV prevention methods were 1.66 times more utilised the services compared to their counterparts (AOR: 1.66, 95% CI: 1.34–2.06). This study was supported by a study done in Addis Ababa and East Hararge [21, 23]. This similarity might be due to having information about the impact of HIV and its preventive method including PMTCT services and health workers might be getting special training on PMTCT service to counsel women. Another significant finding of this study was that women who had knowledge of MTCT of HIV were 2.69 times more utilised the PMTCT services than those who did not know (AOR:2.69, 95% CI:2.16–3.36). This finding is consistent with a related study done in East Hararge [23] and Amhara region [12, 14].

Furthermore, those rural dwellers were 48% less likely to utilise the PMTCT services compared to those urban dwellers (AOR: 0.52, 95% CI: 0.32–0.85) and supported by a study done in Ethiopia and Mizan Aman [28, 30]; because those rural dwellers have low information access, physical access centralisation (infrastructure, transportation problem), and women empowerment (poor family support).

### Limitation of the study

The study tried to measure PMTCT services’ utilisation during antepartum, intrapartum, and postpartum; however, mothers might experience recall bias, mainly the intrapartum period’s services. The other possible bias might be the social desirability bias because the data collectors were health professionals.

## Conclusions

The PMTCT services utilisation in Ethiopia among women during the antepartum, intrapartum, and postpartum periods was surprisingly low and affected by individual and community level factors. The comprehensive knowledge of mothers improves PMTCT utilisation. The utilisation among poor wealth status and rural dweller women was low. Increasing awareness creation on MTCT/PMTCT for women at the community level through strengthening mother group, health development army, as well as HEWs and providing community-based health education on PMTCT services would increase services utilisation in the country. Moreover, the study finding also indicates that the need for better service accessibility, improving the economic status, and empowering HIV-positive pregnant women by raising PMTCT knowledge, especially among uneducated women, will positively affect PMTCT utilisation. Therefore, the federal and regional health bureau and other implementers should emphasise awareness creation and strengthen the existing community-based health extension program to boost PMTCT service utilisation.

## Data Availability

The data used for the current study will be available upon reasonable requests from the corresponding author.

## Abbreviations

ANC: Antenatal Care
ART: Antiretroviral therapy
CSA: Central Statistical Agency
EDHS: Ethiopian Demographic Health Survey
MTCT: Mother to child transmission
PMTCT: Prevention of Mother to child transmission of HIV
PHC: Primary Health Care
WHO: World Health Organization
